# Delta-Opioid Receptor Analgesia Is Independent of Microglial Activation in a Rat Model of Neuropathic Pain

**DOI:** 10.1371/journal.pone.0104420

**Published:** 2014-08-08

**Authors:** Joanna Mika, Katarzyna Popiolek-Barczyk, Ewelina Rojewska, Wioletta Makuch, Katarzyna Starowicz, Barbara Przewlocka

**Affiliations:** Department of Pain Pharmacology, Institute of Pharmacology, Polish Academy of Sciences, Krakow, Poland; University of Kentucky Medical Center, United States of America

## Abstract

The analgesic effect of delta-opioid receptor (DOR) ligands in neuropathic pain is not diminished in contrast to other opioid receptor ligands, which lose their effectiveness as analgesics. In this study, we examine whether this effect is related to nerve injury-induced microglial activation. We therefore investigated the influence of minocycline-induced inhibition of microglial activation on the analgesic effects of opioid receptor agonists: morphine, DAMGO, U50,488H, DPDPE, Deltorphin II and SNC80 after chronic constriction injury (CCI) to the sciatic nerve in rats. Pre-emptive and repeated administration of minocycline (30 mg/kg, i.p.) over 7 days significantly reduced allodynia and hyperalgesia as measured on day 7 after CCI. The antiallodynic and antihyperalgesic effects of intrathecally (i.t.) administered morphine (10–20 µg), DAMGO (1–2 µg) and U50,488H (25–50 µg) were significantly potentiated in rats after minocycline, but no such changes were observed after DPDPE (10–20 µg), deltorphin II (1.5–15 µg) and SNC80 (10–20 µg) administration. Additionally, nerve injury-induced down-regulation of all types of opioid receptors in the spinal cord and dorsal root ganglia was not influenced by minocycline, which indicates that the effects of opioid ligands are dependent on other changes, presumably neuroimmune interactions. Our study of rat primary microglial cell culture using qRT-PCR, Western blotting and immunocytochemistry confirmed the presence of mu-opioid receptors (MOR) and kappa-opioid receptors (KOR), further we provide the first evidence for the lack of DOR on microglial cells. In summary, DOR analgesia is different from analgesia induced by MOR and KOR receptors because it does not dependent on injury-induced microglial activation. DOR agonists appear to be the best candidates for new drugs to treat neuropathic pain.

## Introduction

Neuropathic pain has been shown in clinical and animal studies to be resistant to alleviation by morphine [1], [2], [3], [4], [5], but the mechanism of this effect is unclear. The classical opioid system modulates nociception through three G-protein-coupled receptors: delta-opioid receptors (DOR) [6], [7] kappa-opioid receptors (KOR) [8], [9] and mu-opioid receptors (MOR) [8]. Opioid receptors do not necessarily function independently and can exist as heterodimers that modulate their pharmacology [10], [11], [12]. Several experimental studies have emphasised that the effects of DOR agonists are distinctively more potent than those of MOR and KOR receptors [13], [14], [15] in neuropathic pain. The field of DOR analgesia has been widely studied [16], [17], [18] and the DOR agonists seems to be a good drugs that would be effective in neuropathic pain, but still some of the aspects of DOR ligands interactions have to be clarified.

Reduction of morphine antinociceptive potency has been postulated to be a consequence of changes in the activity of opioid systems or opioid-specific signalling [3], [19]. Although a reduction in the number of receptors may be a major factor in the reduced efficacy of opioids, it has become clear that many other factors affect the efficacy of morphine. Such factors include heterologous desensitisation between opioid and proinflammatory chemokine receptors via shared G-protein-coupled systems [20], down-regulation of glutamate transporters in glial cells [21] or release of such substances as NO, ATP, excitatory amino acids, prostaglandins, and proinflammatory cytokines from activated glia [22], [23], [24], [25], [26], [27]. In a previous study, we used minocycline, which is a tetracycline derivative with pleiotropic biological effects, to clarify whether the analgesic opioid effect is associated with the activation of microglia. Minocycline is a potent inhibitor of the microglial activation [22], [28], [29], [30] that impairs microglial viability and migration [31], [32]. Minocycline also up-regulates a tissue inhibitors of matrix metalloproteinases (TIMPs) [33], inhibits MMP-9 [34] and has been shown in many studies to have neuroprotective effects [35], [36]. Some authors have suggested that minocycline may also reduce pain by inhibiting Ca^2+^ and Na^+^ currents in sensory neurons in DRG [35], [37]. It was shown that pain after nerve injury depended on activation of the p38 MAPK signalling pathway in the spinal cord and p38 MAPK was co-localized with activated microglia [38], [39]. It was shown [40], [41], [42] that the level of phospho-p38 MAPK in microglia was reduced after minocycline treatment, which suggests that this kinase is a minocycline target. Little is known about its influence on neurons, but some authors suggest its neuroprotective effects in dopaminergic and glutamatergic neurons [43], [44]. We demonstrated in our previous study that chronic administration of minocycline attenuated the development of neuropathic pain and enhanced morphine analgesia [45], [46], [47]. Filipovic and Zecevic [48] demonstrated in *in vitro* studies that minocycline protects neurons from LPS-induced inflammation by inhibiting the activation of microglia. Activated microglia may weaken morphine analgesia by releasing pronociceptive factors, and the administration of minocycline antagonises this weakening [23], [46], [47].

The aim of the present study was to determine the extent to which the activation of microglia induced by injury to the sciatic nerve (by chronic constriction injury, CCI) in rats changes the analgesic effect of opioid receptors agonists. Minocycline, a well characterized drug for inhibiting microglial activity [49], was intraperitoneally (i.p.) administered pre-emptively and repeatedly to CCI-exposed rats, subsequently its influence on allodynia and hyperalgesia was measured. On day 7 after CCI, vehicle-treated and minocycline-treated rats received the opioid receptor agonists morphine, DAMGO, U50,488H, DPDPE, deltorphin II and SNC80 intrathecally. We conducted behavioural experiments and simultaneously analysed molecular changes in the transcription of *MOR*, *KOR*, and *DOR* mRNA in the dorsal horn of the lumbar spinal cord and in DRG (L4–L6) at day 7 after injury in CCI-exposed rats after minocycline or vehicle administration. We also analysed the presence of MOR, KOR and DOR in rat primary microglial cell cultures by using qRT-PCR, Western blotting and immunocytochemistry.

## Methods

### Animals

Male Wistar rats (200–350 g) were housed in cages that were lined with sawdust under a standard 12/12 h light/dark cycle (lights on at 08:00 h), with food and water available *ad lib*. Care was taken to reduce the number of animals used, and all experiments were performed according to the recommendations of IASP [50] and the NIH Guide for the Care and Use of Laboratory Animals and were approved by the II Local Bioethics Committee branch of the National Ethics Committee for Experiments on Animals based at the Institute of Pharmacology, Polish Academy of Sciences (Cracow, Poland).

### Surgical preparations

Chronic constriction injury (CCI) was produced in rats by tying four ligatures around the sciatic nerve under sodium pentobarbital anaesthesia (60 mg/kg; i.p.). The *biceps femoris* and the *gluteus superficialis* were separated, and the right sciatic nerve was exposed. The ligatures (4/0 silk) were tied loosely around the nerve distal to the sciatic notch with 1-mm spacing until they elicited a brief twitch in the respective hind limb. The procedure has been described in detail by Bennett and Xie, [51]. After the surgery, all rats developed long-lasting neuropathic pain symptoms such as allodynia and hyperalgesia. Because we have shown in earlier studies that there are no differences between the nociceptive responses of naïve and sham rats [52], we used naïve animals for the behavioural experiments in the present study.

### Intrathecal (i.t.) injection

Rats were prepared for intrathecal (i.t.) injection by implanting catheters under pentobarbital anaesthesia (60 mg/kg i.p.). The intrathecal catheter consisted of polyethylene tubing that was 12 cm long (PE 10, Intramedic; Clay Adams, Parsippany, NJ) with an outside diameter of 0.4 mm and a dead space of 10 µl that had been sterilised by immersion in 70% (v/v) ethanol and fully flushed with sterile water before insertion. Rats were placed on a stereotaxic table (David Kopf Instruments, Tujunga, CA), and an incision was made in the atlanto-occipital membrane. The catheter (7.8 cm of its length) was carefully introduced into the subarachnoid space at the rostral level of the spinal cord lumbar enlargement (L4–L5) according to the method of Yaksh and Rudy [53]. After the implantation, the first injection of 10 µl of water was performed slowly and the catheter was tightened. One day after catheter implantation, the rats were monitored for physical impairments. Those showing motor deficits were excluded from further study. Animals were allowed a minimum 1 week of recovery after the surgery before the experiment began. Water for injection or respective drugs were delivered slowly (1–2 min) in a volume of 5 µl through the i.t. catheter and were followed by 10 µl of water for injection, which flushed the catheter.

### Behavioural tests

#### Tactile allodynia (von Frey test)

Allodynia was measured in rats subjected to CCI by the use of an automatic von Frey apparatus (Dynamic Plantar Aesthesiometer Cat. No. 37400, Ugo Basile Italy). Rats were placed in plastic cages with a wire net floor 5 min before the experiment. The von Frey filament was applied to the midplantar surface of the hind foot, and measurements were taken automatically [23]. The strength of the von Frey stimuli ranged from 0.5 to 26 g in rats.

#### Cold hyperalgesia (cold plate test)

Hyperalgesia was assessed using the cold plate test (Cold/Hot Plate Analgesia Meter No. 05044 Columbus Instruments, USA) as has been described previously [23], [45]. The temperature of the cold plate was maintained at 5°C, and the cut-off latency was 30 s. The rats were placed on the cold plate, and the time until lifting of the hind foot was recorded. The injured foot was the first to react in every case.

### Drug administration

**Figure 1:**
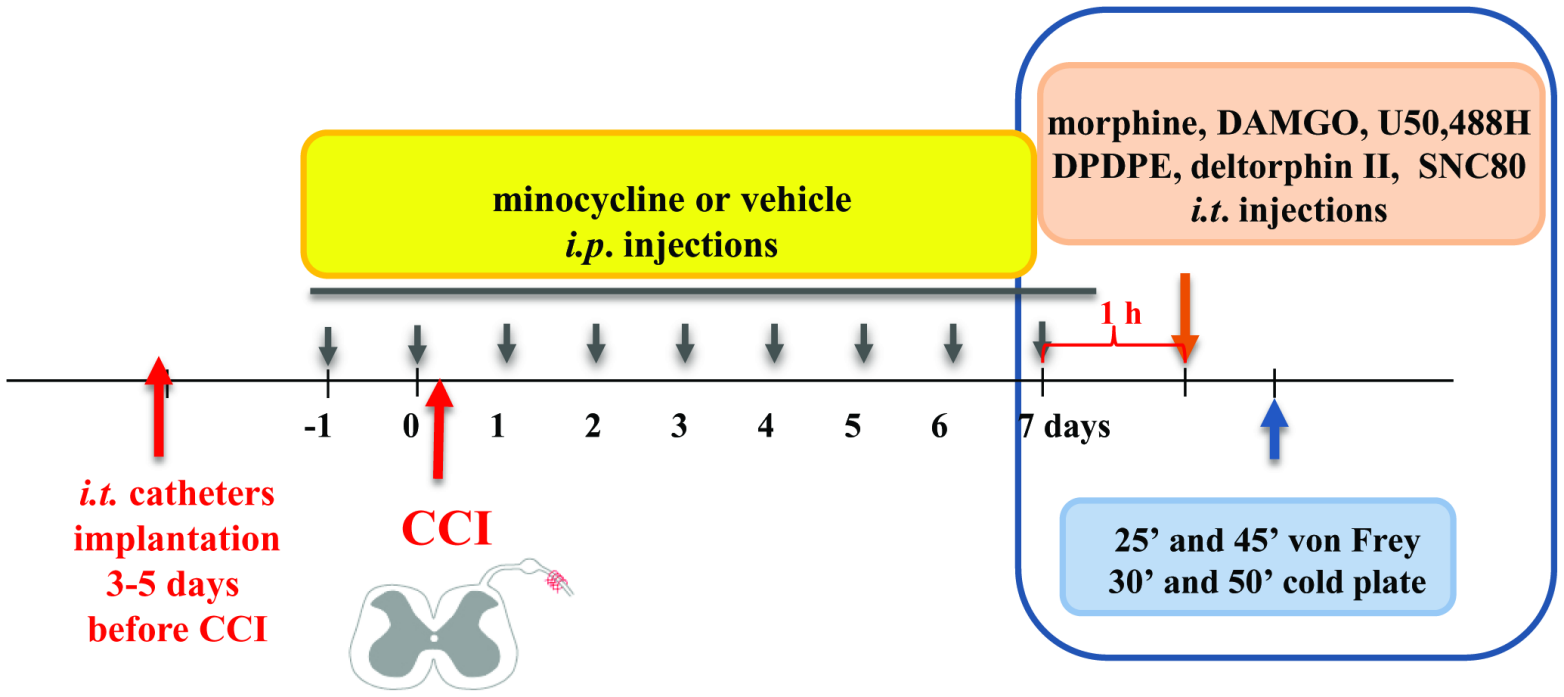
Drug administration.

The chemicals used in this study and their sources were as follows: morphine hydrochloride – Polfa (Kutno, Poland); DAMGO (Sigma-Aldrich, USA), Deltorphin II (Sigma-Aldrich, USA), DPDPE (Sigma-Aldrich, USA), U50,488H (Tocris, UK), SNC80 (Sigma-Aldrich, USA) and minocycline hydrochloride (Sigma-Aldrich, USA). Minocycline (MC; 30 mg/kg; i.p.) was dissolved in sterile water and pre-emptively administered intraperitoneally 16 h and 1 h before CCI and then twice daily for 7 days. This method of minocycline administration was used throughout the work and is referred to in the text as “repeated administration”. The control groups received a vehicle (water for injection) according to the same schedule. One hour after the last morning of minocycline or vehicle administration on day 7 after CCI, morphine (M; 20; 40 µg; 62.3 nM; 124.6 nM), DAMGO (MOR selective ligand; 1; 2 µg; 1.94 nM; 3.9 nM), U50,488H (KOR selective ligand; 25; 50 µg; 61.6 nM; 123.2 nM), DPDPE (DOR I selective ligand; 10; 20 µg; 15.48 nM; 30.96 nM), deltorphin II (del II; DOR II selective ligand; 1.5; 15 µg; 1.9 nM; 19 nM), SNC80 (10; 20 µg; 22.24 nM; 44.48 nM) or a vehicle was intrathecally injected. The von Frey test (25 and 45 min later) and cold plate tests (30 and 50 min later) were carried out after vehicle or opioid agonist administration (Figure 1).

### Microglial cell cultures and treatments

Primary cultures of microglial cells were prepared from 1-day-old Wistar rat pups as has been previously described [54] Briefly, cells were isolated from the rats' cerebral cortices and were plated at a density of 3×10^5^ cells/cm^2^ in a culture medium that consisted of DMEM/Glutamax/high glucose (Gibco, USA) supplemented with heat-inactivated 10% foetal bovine serum (Gibco, USA), 100 U/ml penicillin, and 0.1 mg/ml streptomycin (Gibco, USA) on poly-L-lysine coated 75 cm^2^ culture flasks and were maintained at 37°C and 5% CO_2_. The culture medium was changed after 4 days. The loosely adherent microglial cells were recovered after 9 days by mild shaking and centrifugation. Microglial cells were suspended in a culture medium and plated at a final density of 2×10^5^ cells onto 24 well plates and 1.2×10^6^ cells onto 6 well plates. Adherent cells were incubated for 48 h in a culture medium before being used for the analyses. Cell specificity was determined using an antibody to OX-42 (microglial marker) in cultures of primary microglia. Levels of mRNA for *C1q* (a microglial marker) and *GFAP* (an astroglial marker) were also investigated. Cultured primary microglia were more than 95% positive for OX-42 and C1q.

We have also analyzed the level of MOR, DOR and KOR mRNA level after LPS-stimulation (data not shown). Cells were prepared as described above and then incubated for 6 h with LPS [100 ng/ml] [55] or vehicle (PBS). We have not observed any significant changes in expression of MOR and KOR transcripts in comparison with vehicle-treated cells. In the LPS-stimulated microglial cells the presence of *DOR* mRNA also haven't been detected. Lipopolysaccharides from *Escherichia coli* 0111:B4 (Sigma-Aldrich, USA).

### qRT-PCR analysis of gene expression

Total RNA was extracted according to the method Chomczynski and Sacchi [56] using the TRIzol reagent (Invitrogen) as previously described [57]. The RNA concentration was measured using a NanoDrop ND-1000 Spectrometer (NanoDrop Technologies). Reverse transcription was performed on 500 ng (from cell cultures) or 1000 ng (from tissue) of total RNA using Omniscript reverse transcriptase (Qiagen Inc.) at 37°C for 60 min. cDNA was diluted 1∶10 with H_2_O. qRT-PCR was performed using Assay-On-Demand TaqMan probes according to the manufacturer's protocol (Applied Biosystems) and run on a Real-Time PCR iCycler device (BioRad, Hercules, CA, USA). Rn00561699_m1 (*Oprd1*), Rn01430371_m1 (*Oprm1*), and Rn00567737_m1 (*Oprk1*) were used as TaqMan primers and probes. The expression of HPRT (a housekeeping gene) was quantified to control for variation in cDNA amounts. Cycle threshold values were calculated automatically by iCycler IQ 3.0 software with default parameters. Abundance of RNA was calculated as 2^−(threshold cycle)^.

### Western blot analysis

Cell lysates were collected in a RIPA buffer with a protease inhibitor cocktail and cleared by centrifugation (14000×g for 30 min, 4°C). Samples containing 20 µg of protein were heated in a loading buffer (50 mM Tris–HCl, 2% SDS, 2% β-mercaptoethanol, 4% glycerol and 0.1% bromophenol blue) for 5 min at 70°C and resolved by SDS–PAGE on 12% polyacrylamide gels. Following gel electrophoresis, the proteins were electrophoretically transferred to Immune-Blot PVDF membranes (Bio-Rad). The blots were blocked for 30 min using 5% non-fat dry milk (Bio-Rad) in Tris-buffered saline with 0.1% Tween 20 (TBST). The blots were incubated with primary antibodies (rabbit polyclonal anti-MOR, 1∶500 [58]; rabbit polyclonal anti-DOR, 1∶500 [59]; rabbit polyclonal anti-KOR 1∶500, Abcam [58] that had been diluted in a SignalBoost Immunoreaction Enhancer Kit (Merck Millipore) for 24 h at 4°C and then incubated with a goat polyclonal antibody that had been conjugated to horseradish peroxidase (goat anti-rabbit IgG, BioRad) at a dilution of 1∶1000 for 1 h at room temperature. After four 5-minute washes in TBST, immunocomplexes were detected using a Lumi-Light Western Blotting Kit and visualised using a Fujifilm LAS-4000 fluorimager system. The blots were washed 4 times for 5 minutes each in TBST and reprobed with a mouse antibody against GAPDH (1∶5000, Millipore) as a loading control. The relative levels of immunoreactivity were quantified using Fujifilm Image Gauge software.

### Immunocytochemical analysis

We used commercially available specific anti-MOR, anti-KOR and anti-OX/42 antibodies. Cells were fixed for 20 minutes in 4% paraformaldehyde in a 0.1 M phosphate buffer (pH 7.4) and incubated with primary antibodies (rabbit anti-MOR, 1∶400 [60], rabbit anti-KOR, 1∶400 [61], rabbit anti-DOR, 1∶400, [62] Neuromics; mouse anti-OX/42, 1∶500, Serotec) for 2 days at 4°C. After three washes in PB, double immunofluorescence was revealed by incubation for 2 h in the appropriate fluorochrome-conjugated secondary antibody, Alexa Fluor546 donkey anti-rabbit and Alexa Fluor488 donkey anti-mouse, diluted 1∶500 in 5% NDS. Sections were then washed with PB and coverslipped with an Aquatex mounting medium (Merck, Darmstadt, Germany). Sections without primary antibodies were used as negative controls.

### Data analysis

The behavioural data are presented as the mean ± S.E.M of 8–16 rats per group. The results of the experiments were statistically evaluated using one-way analysis of variance (ANOVA). All of the differences between the treatment groups were further analysed with Bonferroni's post-hoc tests. Significant differences in comparisons with vehicle-treated CCI-exposed rats are indicated by *P (<0.05), **P (<0.01) and ***P (<0.001). Significant differences between vehicle-treated CCI-exposed rats that had received a single dose of opioid receptor ligands and minocycline-treated CCI-exposed rats that had received a single dose of opioid receptor ligands are indicated by ^#^ *P* (<0.05), ^##^ *P* (<0.01) and ^###^ *P* (<0.001).

The qRT-PCR analyses were performed in three groups: naïve, CCI-exposed and minocycline-treated CCI-exposed rats. The results are presented as fold changes compared with the naïve rats in the ipsilateral dorsal lumbar spinal cord and DRG. The qRT-PCR data are presented as the mean ± S.E.M and represent the normalised averages that were derived from the threshold qRT-PCR cycles from four to eight samples for each group. Intergroup differences were analysed using ANOVAs followed by Bonferroni's multiple comparison tests. Significant differences resulting from comparison with naïve rats are indicated by *P (<0.05), **P (<0.01) and ***P (<0.001). The data from 3 to 10 cell cultures are presented.

## Results

### Repeated administration of minocycline significantly diminished allodynia and hyperalgesia in neuropathic pain in rats

**Figure 2:**
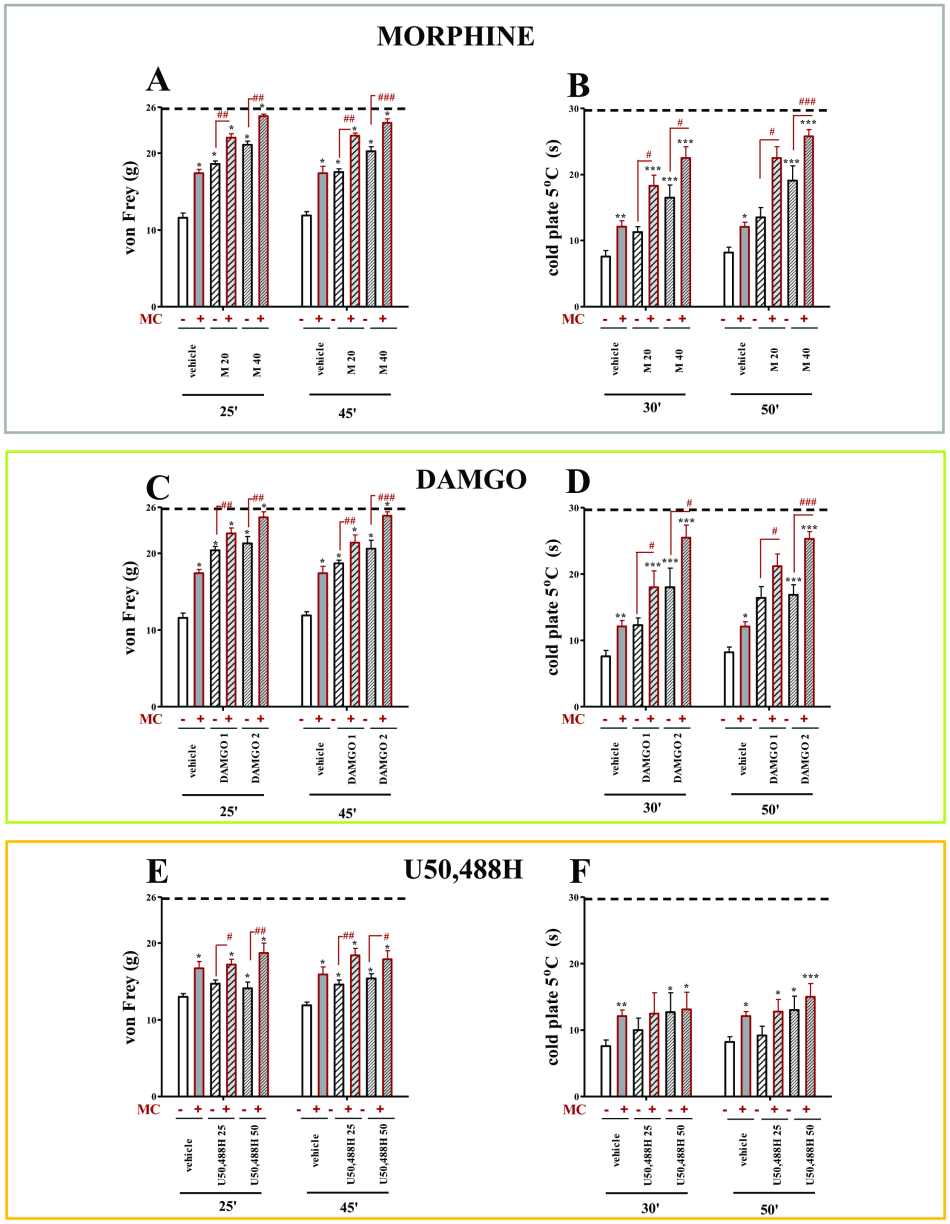
The effects of morphine, DAMGO and U50,488H on vehicle- and minocycline-treated CCI-exposed rats. The response to morphine, DAMGO and U50,488H was measured 25 and 45(A, C, E) and 30 and 50 minutes after administration by the cold plate test (B, D, F). Minocycline (MC; 30 mg/kg; i.p.) was administered intraperitoneally pre-emptively 16 h and 1 h before CCI, and then repeatedly twice daily for 7 days. Vehicle-treated and minocycline-treated rats received intrathecal morphine (M; 20; 40 µg/5 µl), DAMGO (1; 2 µg/5 µl) or U50,488H (25; 50 µg/5 µl) one hour after the last morning administration on day 7 after CCI. The data are presented as the mean response ± S.E.M. (8–16 rats per group). The results of the experiments were statistically evaluated using One-way Analyses of Variance (ANOVA). The differences between the treatment groups throughout the study were further analysed with Bonferroni's *post-hoc* tests. *P<0.05, **P<0.01 and ***P<0.001 indicate significant differences compared with vehicle-treated CCI-exposed rats; #*P*<0.05, ##*P*<0.01 and ###*P*<0.001 indicate significant differences between vehicle-treated CCI-exposed rats that received a single dose of morphine and minocycline-treated CCI-exposed rats that received a single dose of morphine, DAMGO or U50,488H. Dotted line is a value for naïve animals (for von Frey test 25.8 g; for cold plate test 29.7 s).

**Figure 3:**
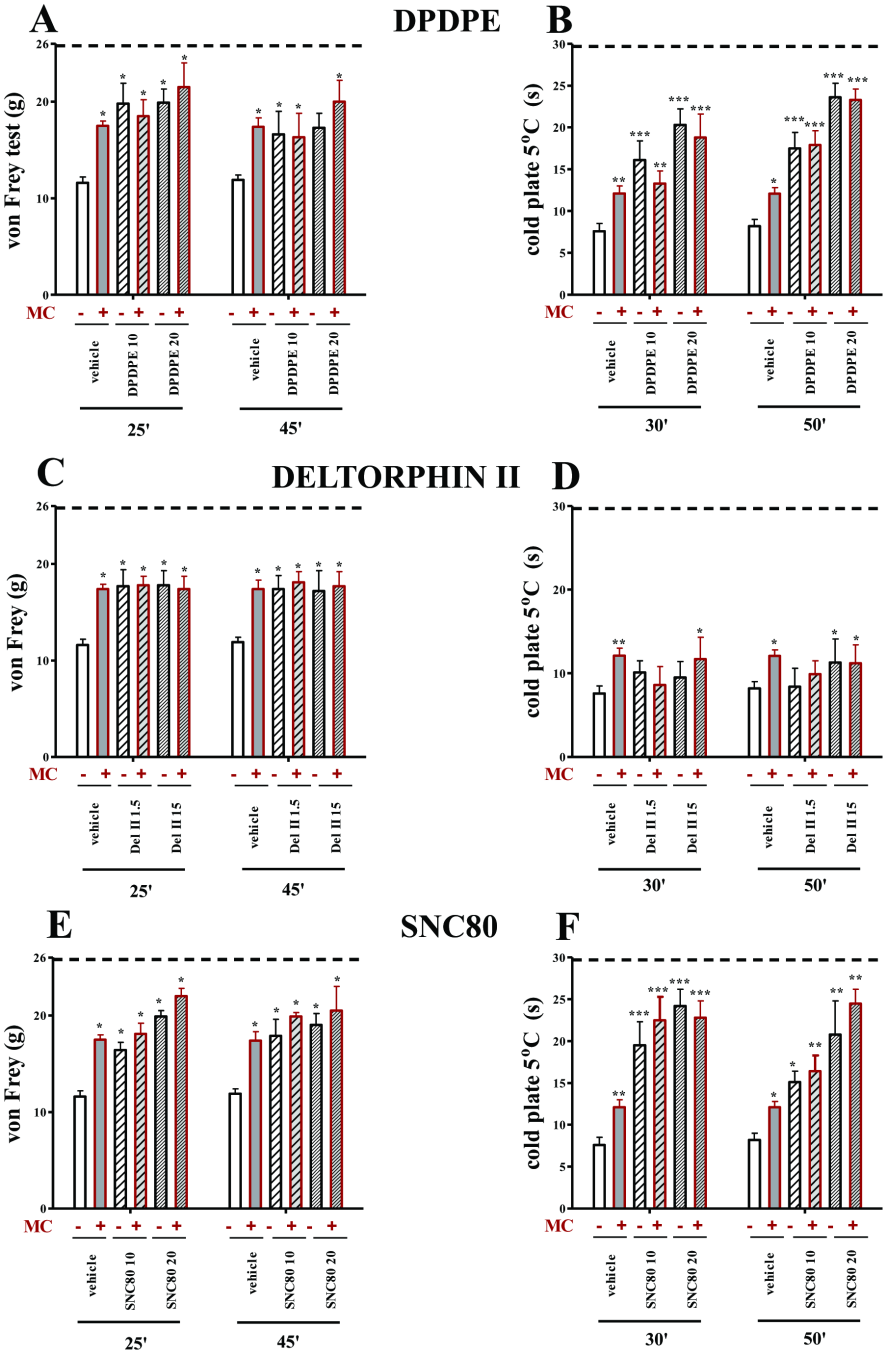
The effects of DPDPE, deltorphin II and SNC80 on vehicle- and minocycline-treated CCI-exposed rats. The response to DPDPE, deltorphin II and SNC80 was measured 25 and 45(A, C, E) and 30 and 50 minutes after administration by the cold plate test (B, D, F). Minocycline (MC; 30 mg/kg; i.p.) was administered intraperitoneally pre-emptively 16 h and 1 h before CCI, and then repeatedly twice daily for 7 days. Vehicle-treated and minocycline-treated rats received intrathecal DPDPE (10; 20 µg/5 µl), deltorphin II (del II; 1.5; 15 µg/5 µl) or SNC80 (10; 20 µg/5 µl) one hour after the last morning administration on day 7 after CCI. The data are presented as the mean response ± S.E.M. (8–16 rats per group). The results of the experiments were statistically evaluated using ANOVAs. The differences between the treatment groups throughout the study were further analysed with Bonferroni's *post-hoc* tests. *P<0.05, **P<0.01 and ***P<0.001 indicate significant differences compared with vehicle-treated CCI-exposed rats. Dotted line is a value for naïve animals (for von Frey test 25.8 g; for cold plate test 29.7 s).

All vehicle-treated CCI rats exhibited neuropathic pain symptoms in the behavioral tests. The rats exhibited strong allodynia on the seventh day after ligation as measured by the von Frey test (11.6 g±0.6 vs. 25.8 g±0.2 for naïve rats) (Fig. 2A,C,E; 3A,C,E) and potent hyperalgesia as measured by the cold plate test (7.6 s±0.9 vs. 29.7 s±0.3 for naïve rats) (Fig. 2B,D,F; 3B,D,F). Repeated administration of minocycline (MC; 30 mg/kg; i.p.) attenuated allodynia (17.4 g±0.5 vs. 11.6 g±0.6 for the vehicle-treated CCI rats) (Fig. 2A,C,E; 3A,C,E) and also hyperalgesia (12.1 s±0.9 vs. 7.6 s±0.9 for the vehicle-treated CCI rats) (Fig. 2B,D,F; 3B,D,F) to a similar extent at both time points.

### Repeated administration of minocycline significantly influenced the effects of morphine in neuropathic pain in rats

The doses of morphine effective in neuropathic pain (M; 20 and 40 µg; i.t.) were used. Morphine was injected one hour after the last morning dose of minocycline (30 mg/kg; i.p. repeatedly) or vehicle, and allodynia and hyperalgesia were measured (see Figure 1). Morphine at a dose of 20 µg significantly diminished allodynia 25 and 45 minutes after injection compared with vehicle-treated animals (18.60 g±0.4 vs. 11.6 g±0.6 and 17.57 g±0.39 vs. 11.9 g±0.5, respectively; Fig. 2A). In minocycline-treated animals, the antiallodynic effect of morphine at a dose of 20 µg was potentiated by minocycline at both times as shown in the von Frey test (25 minutes: 22.04 g±0.52 vs. 18.60 g±0.4 for morphine-treated rats, 45 minutes: 22.3 g±0.34 vs. 17.57 g±0.39 for morphine-treated rats; Fig. 2A). Morphine at a dose of 20 µg did not influence thermal hyperalgesia but in the group which was injected repeatedly with minocycline, the antihyperalgesic effect of morphine was demonstrated (30 minutes: 18.30 s±1.6 vs. 11.3 g±0.8 for morphine-treated rats, 50 minutes: 22.5 s±1.7 vs. 13.5 s±1.5 for morphine-treated rats; Fig. 2B). A higher dose of morphine (40 µg) was effective in reducing mechanical allodynia 25 and 45 minutes after injection compared with vehicle-treated animals (25 minutes: 21.1 g±0.48 vs. 11.6 g±0.6, 45 minutes: 23.9 g±0.6 vs. 11.9 g±0.5; Fig. 2A). In minocycline-treated rats, the antiallodynic effect of morphine at a dose of 40 µg was potentiated at both times (25 minutes: 24.84 g±0.27 vs. 21.1 g±0.48, 45 minutes: 23.98 g±0.51 vs. 20.25 g±0.6; Fig. 2A). In opposite to the lower dose, morphine at a dose of 40 µg showed antihyperalgesic effect at both time points (30 minutes: 16.5 s±1.9 vs. 7.6 s±0.9, 50 minutes: 19.1 s±2.2 vs. 8.2 s±0.8; Fig. 2B). The increased effect of morphine at a dose of 40 µg in minocycline-treated group was observed at both time points, however, this effect was more significant after 50 minutes (25.8 s±1.0 vs. 19.1 s±2.2; Fig. 2B).

### Repeated administration of minocycline significantly influenced the effects of MOR (DAMGO) and KOR (U50,488H) agonists in neuropathic pain in rats

DAMGO at a dose of 1 µg significantly diminished allodynia 25 and 45 minutes after injection compared with vehicle-treated animals (25 minutes: 20.4 g±0.5 vs. 11.6 g±0.6, 45 minutes: 18.7 g±0.4 vs. 11.9 g±0.5; Fig. 2C). In minocycline-treated animals, the antiallodynic effect of DAMGO (1 µg) was potentiated at both times as shown in the von Frey test (25 min: 22.6 g±0.7 vs. 20.4 g±0.5 for DAMGO-treated rats, 45 min: 21. 4 g±1.0 vs. 18.7 g±0.4 for DAMGO-treated rats; Fig. 2C). DAMGO at a dose of 1 µg did not influence thermal hyperalgesia but in minocycline- and DAMGO-treated group the antyhiperalgesic effect occurred at both time points but the better effect we observed 50 min after injection (21.2 s±1.8 vs. 16.4 s±1.7 for DAMGO-treated rats; Fig. 2D). The higher dose of DAMGO (2 µg) was effective in reducing mechanical allodynia 25 and 45 minutes after injection compared with vehicle-treated animals (25 min: 21.3 g±0.9 vs. 11.6 g±0.6, 45 min: 20.6 g±1.1 vs. 11.9 g±0.5; Fig. 2C). As shown in the von Frey test, repeated minocycline administration potentiated effect of DAMGO at a dose of 2 µg at both times, but a better effect was present after 45 minutes (24.9 g±0.5 vs. 20.6 g±1.1; Fig. 2C). In opposite to the lower dose, DAMGO at a dose of 2 µg showed antihyperalgesic effect at both time points (30 min: 18.0 s±2.9 vs. 7.6 s±0.9, 50 min: 16.9 s±1.5 vs. 8.2 s±0.8; Fig. 2D). We also observed increased effect of DAMGO in minocycline-treated group 30 minutes after opioid injection, however, this effect was more significant after 50 minutes (25.3 s±1.1 vs.16.9 s±1.5; Fig. 2D).

During neuropathy, the antiallodynic effect of U50,488H appeared after doses of 25 µg i.t. only 45 minutes after injection (14.6 g±0.6 vs. 11.9 g±0.5; Fig. 2E). The effect of this dose of U50,488H was potentiated in minocycline-treated group as shown in the von Frey test at both times, but at 45 minutes after injection the effect was more pronounced (18.4 g±0.9 vs. 14.6 g±0.6; Fig. 2E). U50,488H at a dose of 25 µg did not influence hyperalgesia and minocycline did not change this action (Fig. 2F). The higher dose (50 µg; i.t.) diminished allodynia at both times (25 min: 14.1 g±0.8 vs. 11.6 g±0.6, 45 min: 15.4 g±0.6 vs. 11.9 g±0.5; Fig. 2E). In minocycline-treated rats, the antiallodynic effect of U50,488H at a dose of 50 µg was potentiated at both times, but it potentiation was higher after 25 minutes (18.7 g±1.3 vs. 14.1 g±0.8; Fig. 2E). This dose of U50,488H slightly diminished hyperalgesia at 30 (12.7 s±2.9 vs. 7.6 s±0.9; Fig. 2F) and 50 (13.0 s±2.1 vs. 8.2 s±0.8; Fig. 2F) minutes after injection. Repeated minocycline administration did not change the action of U50,488H at a dose of 50 µg (Fig. 2F).

### Repeated administration of minocycline did not influence the effects of DOR agonists (DPDPE, Deltorphin II and SNC80) in neuropathic pain in rats

DPDPE at a dose of 10 µg diminished allodynia 25 and 45 minutes after injection compared with vehicle-treated animals (25 min: 19.8 g±2.1 vs. 11.6 g±0.6, 45 min: 16.6 g±2.4 vs. 11.9 g±0.5; Fig. 3A) and minocycline treatment did not change this effect (Fig. 3A). Higher dose of DPDPE (20 µg) was effective in reducing mechanical allodynia only 25 minutes after injection compared with vehicle-treated animals (20.3 g±1.9 vs. 11.6 g±0.6, Fig. 3A) and this antiallodynic effect was not changed by minocycline (Fig. 3A). As shown in the cold plate test, DPDPE significantly diminished thermal hyperalgesia after both doses of 10 and 20 µg (30 min: 16.1 s±2.3 vs. 7.6 s±0.9; 50 min: 17.50 s±1.9 vs. 8.2 s±0.8 and 30 min: 20.3 s±1.9 vs. 7.6 s±0.9; 50 min: 23.6 s±1.7 8.2 s±0.8; respectively; Fig. 3B) and minocycline did not modulate this action (Fig. 3B).

Deltorphin II at a dose of 1.5 µg slightly diminished allodynia 25 and 45 minutes after injection compared with vehicle-treated animals (25 min: 17.7±1.7 vs. 11.6 g±0.6, 45 min: 17.4 g±1.4 vs. 11.9 g±0.5; Fig. 3C) and no changes in deltorphin II action in minocycline-treated group were observed (Fig. 3C). The higher dose of Deltorphin II (15 µg) was effective in reducing mechanical allodynia 25 (17.8 g±1.5 vs. 11.6 g±0.6; Fig. 3C) and 45 (17.2 g±2.1 vs. 11.9 g±0.5; Fig. 3C) minutes after injection compared with vehicle-treated animals. Repeated minocycline injection did not change the antiallodynic effect of deltorphin II (Fig. 3D). Deltorphin II at a dose of 1.5 µg did not influence hyperalgesia in CCI-exposed rats and minocycline also did not change this effect (Fig. 3D). As shown in the cold plate test, deltorphin II at a dose of 15 µg diminished thermal hyperalgesia only 50 minutes after injection (11.3 s±2.8 vs. 8.2 s±0.8; Fig. 3D) and minocycline did not change the antihyperalgesic action of the higher dose of Deltorphin II (Fig. 3D).

SNC80 at a dose of 10 µg slightly diminished allodynia 25 and 45 minutes after injection compared with vehicle-treated animals (25 min: 16.4 g±0.8 vs. 11.6 g±0.6, 45 min: 17.9 g±1.7 vs. 11.9 g±0.5; Fig. 3E). The higher dose of SNC80 (20 µg) was effective in reducing mechanical allodynia 25 (19.9 g±0.6 vs. 11.6 g±0.6; Fig. 3E) and 45 (19.0 g±1.2 vs. 11.9 g±0.5; Fig. 3E) minutes after injection compared with vehicle-treated animals. Repeated minocycline injection did not change the antiallodynic effect of both doses of SNC80 (Fig. 3E). As shown in the cold plate test, SNC80 at a dose of 10 µg influenced hyperalgesia in CCI-exposed rats (30 min: 19.5 s±2.8 vs. 7.6 s±0.9; 50 min: 13.0 s±2.3 vs. 8.2 s±0.8; Fig. 3F) SNC80 at a dose of 20 µg diminished thermal hyperalgesia 30 (24.2 s±2 vs. 7.6 s±0.9; Fig. 3F) and 50 (20.8 s±4.0 vs. 8.2 s±0.8; Fig. 3F) minutes after injection. Minocycline did not change the antihyperalgesic action of both doses of SNC80 (Fig. 3F).

### Repeated administration of minocycline did not influence *MOR*, *DOR* and *KOR* mRNAs during neuropathic pain in rats

**Figure 4:**
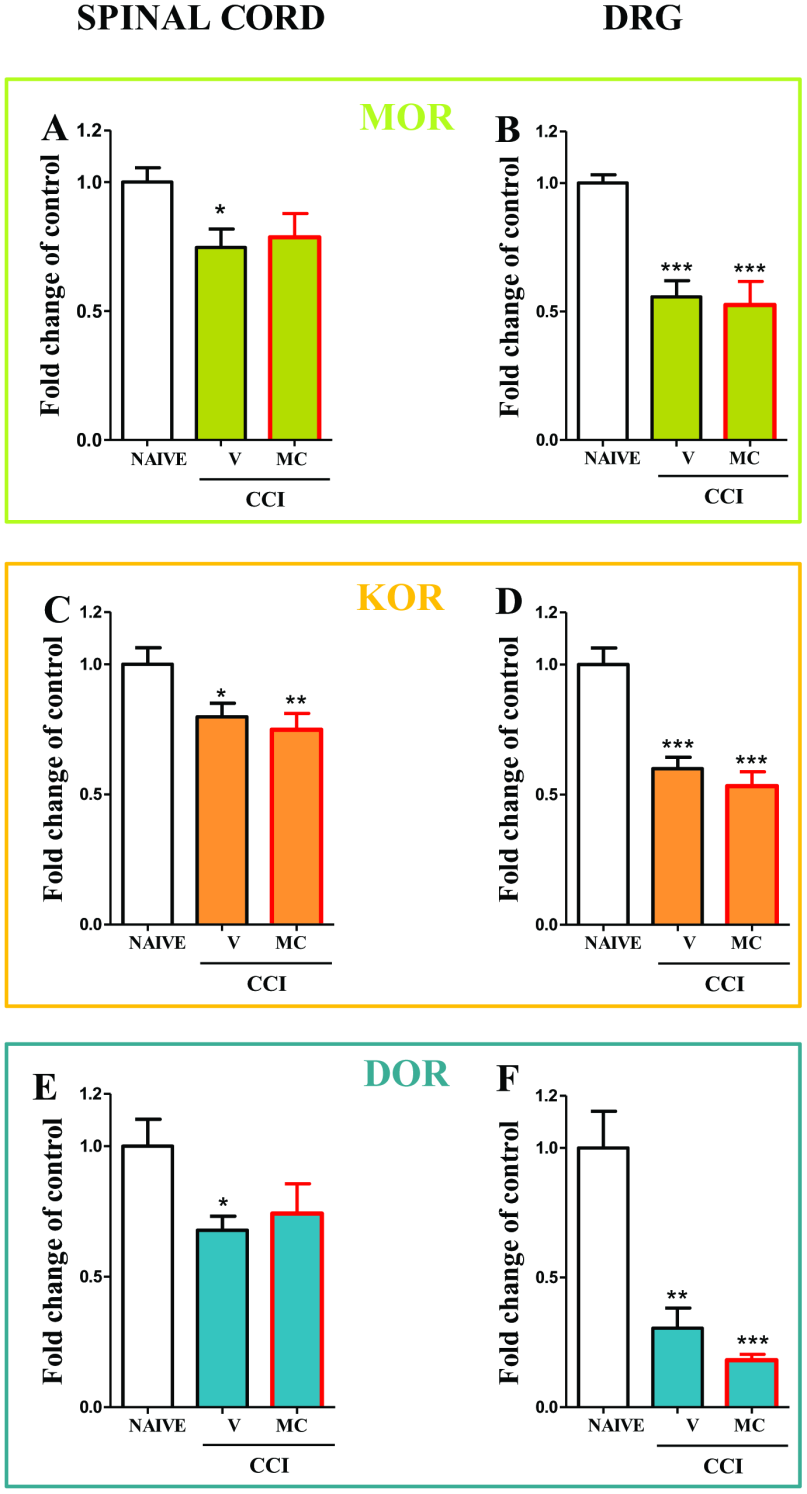
*MOR*, *DOR*, *KOR* mRNAs in spinal cord and DRGs in vehicle- and minocycline-treated CCI-exposed rats. Minocycline (MC; 30 mg/kg; i.p.) was administered intraperitoneally pre-emptively 16 h and 1 h before CCI, and then repeatedly twice daily for 7 days. On the seventh day, spinal cords (L4–L6) and DRG were collected for the qRT-PCR analysis of *MOR* (A, B), *KOR* (C, D) and *DOR* (E, F) gene expression. The data are presented as the means ± SEM and represent the normalised averages derived from the threshold qRT-PCR cycles from four to eight samples for each group. Intergroup differences were analysed using ANOVAs followed by Bonferroni's multiple comparison tests. *P<0.05; **P<0.01; ***P<0.001 indicate significant differences compared with naïve rats.

In the spinal cord the downregulation of *MOR* mRNA from 1±0.05 to 0.7±0.07 (Fig. 4A) and in the DRG from 1±0.03 to 0.5±0.06 (Fig. 4B) was observed compared to the naïve rats. Minocycline not influence the level of *MOR* mRNA in the spinal cord and in the DRG (Fig. 4A and B, respectively).

In the spinal cord the downregulation of *KOR* mRNA from 1±0.06 to 0.8±0.05 (Fig. 4C) and in the DRG from 1±0.06 to 0.6±0.04 (Fig. 4D) was observed compared to the naïve rats. Minocycline not influence the level of *KOR* mRNA in the spinal cord and in the DRG (Fig. 4C and D, respectively).

In the spinal cord the downregulation of *DOR* mRNA from 1±0.1 to 0.67±0.05 (Fig. 4E) and in the DRG from 1±0.1 to 0.3±0.07 (Fig. 4F) was observed compared to the naïve rats. Minocycline not influence the level of *KOR* mRNA in the spinal cord and in the DRG (Fig. 4E and F, respectively).

### Opioid receptor expression in primary microglial cell cultures

**Figure 5:**
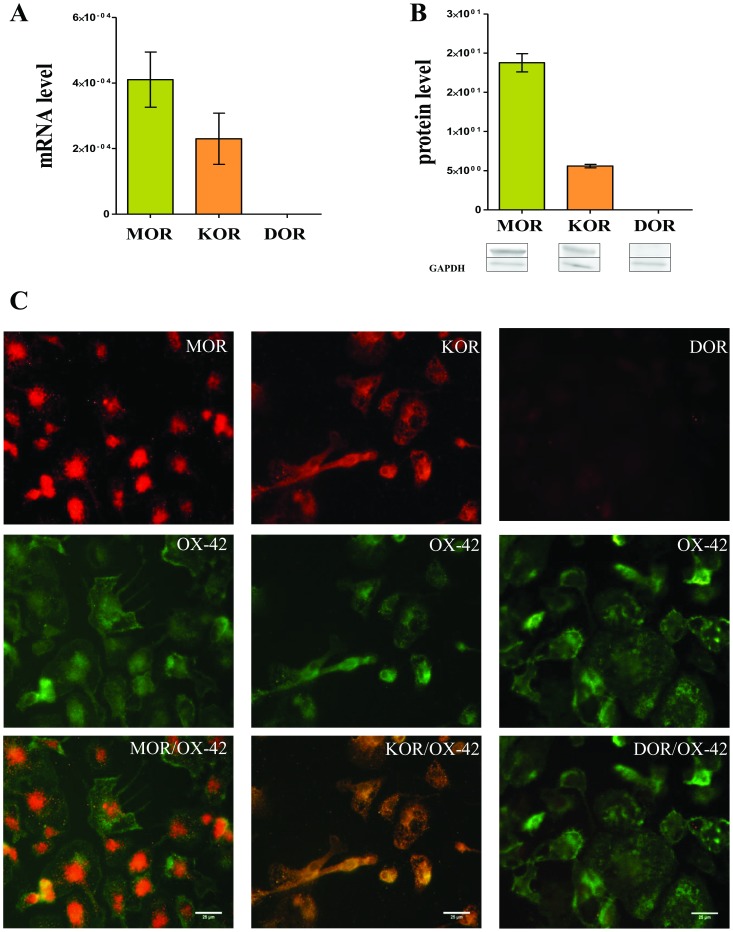
The presence of opioid receptors on microglia cells - *in vitro* studies. We analysed the presence of opioid receptors in primary microglial cell cultures. Using reverse transcriptase-polymerase chain reaction, we found that mRNA for *MOR* and *KOR*, but not *DOR*, is expressed in rat primary microglial cells cultures (A). Western blot analysis (B) detected proteins for MOR and KOR, but not DOR, in microglia. The presence of MOR and KOR, but not DOR, were confirmed by immunocytochemistry (C), and we show the colocalisation of MOR (left panel) and KOR (middle panel) antibodies (red) with OX/42 antibodies (green). The scale bar for all microphotographs is 25 µm. The data from 3–10 cell cultures are presented.

Using a reverse transcriptase-polymerase chain reaction, we found that mRNA for *MOR* and *KOR*, but not for *DOR*, is expressed in rat primary microglial cell cultures (Fig. 5A). In the Western blot analysis, we found that protein for MOR and KOR is present in microglia (Fig. 5B). The protein for DOR was undetectable (Fig. 5B). The expression of MOR and KOR, but not DOR, in microglial cells was confirmed by immunocytochemistry (Fig. 5C).

## Discussion

Opioids are fundamental to the treatment of pain, but their efficacy is limited by side effects, including tolerance and hyperalgesia [63]. Some authors suggest that reduced sensitivity to opioids and the increases in their dosages that are necessary to obtain adequate analgesia heighten the risk of side effects [4], [5], [64]. It remains unclear why morphine is a less potent analgesic in clinical [65], [66] and experimental [15], [46], [67] studies of neuropathic pain in contrast to inflammatory pain [15], [67]. In many studies it was shown that selective DOR agonists do not lose their effectiveness in the treatment of neuropathic pain [13], [15], [68]. It has been suggested that the lesser effects of morphine on neuropathic pain are due to the reduced number of presynaptic opioid receptors that results from nerve damage-induced degeneration of primary afferent neurons [64], [69] besides other effects, like upregulation of the anti-opioid system CCK, and the NMDA receptor-dependent central sensitization, between others. Obara *et al.* [15] used ED_50_ analysis to demonstrate in 2009 that much higher doses of MOR and KOR agonists injected intraplantarly are required to produce antinociception in neuropathic than in inflammatory pain. However, it remains unclear why the ED_50_ of DOR agonists, but not those of MOR and KOR agonists, is comparable in both neuropathic and inflammatory pain [15]. Our study showed that the expression of all types of opioid receptor mRNA in the spinal cord and DRG was decreased in neuropathic pain. Also Stone *et al.* [70], using three animals models of peripheral nerve injury (CCI, SNL and SNT), observed decreases in spinal DOR expression at day 10^th^ after operations at the side of injury. Herradon *et al.* [71] compared two strains of rats, Fischer 344 and Lewis rats, in neuropathic pain model and found significant down-regulation of DOR mRNA 28 days after CCI in the DRG of Lewis rats and the same trend was observed in F344 rats. Those data correspond well with our results obtained at day 7^th^ after CCI in Wistar rats. In the study of Obara *et al.* [15] biochemical experiments were conducted at 3^rd^ and 14^th^ day (but not day 7^th^) after CCI and showed a non-significant, downward trend in the expression of mRNA for *MOR*, *DOR* and *KOR* at the spinal cord level. At the DRG level of neuropathic rats these authors observed down-regulation of mRNA level for *MOR* in L4–L5 DRGs (at day 3^rd^) and in L5 DRG (at day 14^th^), for *DOR* in L5 DRG (at both days) and for *KOR* in L4 and L5 DRGs (at day 3^rd^) and in L5 DRG (at day 14^th^) in comparison with naive animals. Those data correspond well with our results obtained at day 7^th^ after CCI, however, in our experiments we pooled L4–L6 DRGs [15]. In the paper of Kabli and Cahill [68] the up-regulation of DOR protein was observed 14 days after sciatic nerve injury at the DRG level. The authors suggest that the increase in DOR protein is correlated with DOR trafficking to the site of injury, which may explain the lower level of mRNA as shown by our results. Should be noted, however, that in some papers no decrease in opioid receptor expression was observed [71], [72]. Those discrepant data may result from different animal species used in experiments or time of the tissue collection after injury as it is shown in the mentioned works.

However, minocycline does not affect the reduced levels of all opioid receptor mRNAs, although it potentiates analgesia after such opioid agonists as morphine, DAMGO and U50,488H. Therefore, we should consider other mechanisms of weakened analgesia in neuropathic pain that can be restored by minocycline administration. These mechanisms may be important for sustained analgesia after administration of DOR ligands.

Several studies have suggested that the activation of glia and the enhancement of proinflammatory cytokine levels in the spinal cord and DRG are responsible for the development of both neuropathic pain and morphine tolerance [73], [74], [75]. We have previously observed an attenuation of the development of neuropathic pain when we administered minocycline, which is a substance that inhibits microglial activation in CCI-exposed rats [45], [46]. Although minocycline can influence some neuronal functions, its ability to reduce microglial activation [76] as well as its selectivity of the action has been recently demonstrated [49]. We show in the present paper that the antiallodynic and antihyperalgesic effects of morphine, DAMGO and U50,488H, but not of DPDPE, deltorphin II or SNC80, were significantly potentiated with minocycline in CCI-subjected rats. In many studies we may found pharmacological evidence of distinct subtypes of DOR [77]. It is known that DOR_1_ is activated by DPDPE, DOR_2_ by deltorphin II and SNC80 is a a highly selective agonist for DOR. We had demonstrated in our previous studies the occurrence of strong antiallodynic and antihyperalgesic effects of DOR_1_ and DOR_2_ agonists after their acute and chronic i.t. administration in a rat neuropathic pain model [13]. Analgesic effects of i.t. injected SNC80 was also shown in many pain models [77], [78], [79]. In the present paper we have shown that the effectiveness of DPDPE, deltorphin II and SNC80 is not enhanced by minocycline treatment. Our results suggest that DOR analgesia is not dependent on injury-induced microglial activation. DORs are therefore a promising target for the development of analgesics. Indeed as previously reported by Holdridge *et al.* [80], [81] the prolonged morphine treatment-induced incensement in microglial cell size was not functionally relevant in DOR analgesia, which confirms our research concerning the presence of DOR on microglia.

In the *in vitro* study, we used qRT-PCR, Western blot and immunocytochemistry assays to confirm the presence of MOR and KOR in microglia, and we have shown for the first time that those microglial cells do not express DOR. Our results are in agreement with other studies that have shown that microglia express MOR and KOR [82], [83], [84]. Chao *et al.* [85] first reported in 1996 that KOR was present in human microglia using qRT-PCR and a ligand-binding assay. The expression of KOR in microglial cells was also confirmed by the membrane binding of selective ligand [^3^H]U69,593. Additionally, Chao *et al.* 1997 [86] have shown that morphine and DAMGO suppressed human microglia chemotaxis in a dose-dependent fashion and this effect is significantly attenuated by β-funaltrexamine (a MOR selective antagonist), which is consistent with our confirmation of the presence of MOR in microglial cells. The occurrence of DOR in microglial cells is still controversial. Our results strongly suggest that there is no DOR in rat primary microglial cells, although the studies of Turchan-Chlewo *et al.* [87] suggest their presence. However, Turchan-Chlewo *et al.* [87] used the PCR method (Sybr Green), which is not sufficiently selective and can give false positive results. The TaqMan assay that we used is standardised and highly specific, and we confirmed the lack of DOR in microglia cell cultures by using Western blot and immunocytochemistry, therefore, our results are not consistent with those that were obtained by immunostaining by Turchan-Cholewo *et al.* [87] and Thorlin *et al.* [88]. Moreover studies by Turchan-Cholewo *et al.* [87] used commercial antibodies against DOR, which are no longer recommended for such studies. Due to the lack of selective DOR antibody a unique genetic mouse model was developed in order to investigate the distribution of DOR in the nervous system [16], [89]. Opioid receptors have high degrees of homology, and antibodies may recognise other subtypes within the same family. DOR expression has also been observed on glial-like cells in the dentate gyrus [90] and rat cervical spinal cord [91], but none of these authors distinguished between astrocytes and microglia. Our results concerning the absence of DOR in microglia are consistent with recently published results of pharmacological studies that were conducted by Merighi *et al.* [84]. These authors have shown in primary microglia cell cultures that DPDPE, a DOR agonist, does not change the level of protein for PKC after LPS stimulation, while morphine and DAMGO up-regulate this kinase. The ineffectiveness of DPDPE in this study may be explained by the lack of DOR receptors in microglial cells. Horvath and DeLeo, [92] showed that selective agonists of DOR_1_ DPDPE had no effect on microglial migration, which confirms our results that DOR, in contrast to MOR and KOR, is not present in microglial cells. DOR shows a functional profile that is distinct from that of MOR or KOR [93] and plays an important role in chronic pain, for example DOR knockout mice showed augmented neuropathic pain [94], [95]. DOR agonists are poor analgesics in acute pain [96], but are highly effective following inflammatory or neuropathic pain [13], [96], [97], [98], [99].

Happel *et al.* [100] suggested in 2008 that morphine and DAMGO influence the immune system. For example, opioids alter macrophage functions and they modulate cytokine production and chemokine and chemokine receptor expression. Activation of proinflammatory chemokine receptors is known to down-regulate the analgesic functions of opioid receptors, and this enhances the perception of pain [101]. Horvath *et al.* [102] showed that morphine increases microglial migration by means of an interaction between *MOR* and P2X_4_ receptors. This interaction is dependent on PI3K/Akt pathway activation [102]. Under neuropathic pain, the phosphorylation of p38 MAPK in microglia results in increased synthesis of the proinflammatory cytokines IL-1β, IL-6, and TNF-α. Spinal blockade of these cytokines is known to attenuate neuropathic pain and morphine tolerance [74], [103]. Therefore, in cases where MOR and KOR agonists target microglial signalling by inhibiting the actions of chemokines (fractalkine, CCL2), ATP receptors (P2X4, P2X7), MMP-9, p38 MAPK, or/and proinflammatory cytokines (IL-1β, IL-6, and TNF-α) improve their effectiveness. However, this is not the case with DOR agonists. This difference deserves future exploration.

MOR agonists, especially morphine, still remain the drugs of choice for the treatment of neuropathic pain, despite the side effects and limited efficacy. It was shown that DOR agonists were effective in persistent pain [13], [68], [81], [104], [105] and the mechanisms underlying this analgesic action was probably linked with trafficking to the cell membrane or better receptor coupling to signalling effectors [104]. Nadal *et al.* [95] demonstrated that neuropathic pain was enhanced in delta-opioid receptor knockout mice. Some authors suggest a delta opioid agonists as a promising alternative to mu analgesics in the treatment of chronic pain [13], [95], [104], [106]. Currently, research is being conducted on analgesic effects of new DOR ligands [107] and some of these substances are being tested in clinical trials [108].

**Figure 6:**
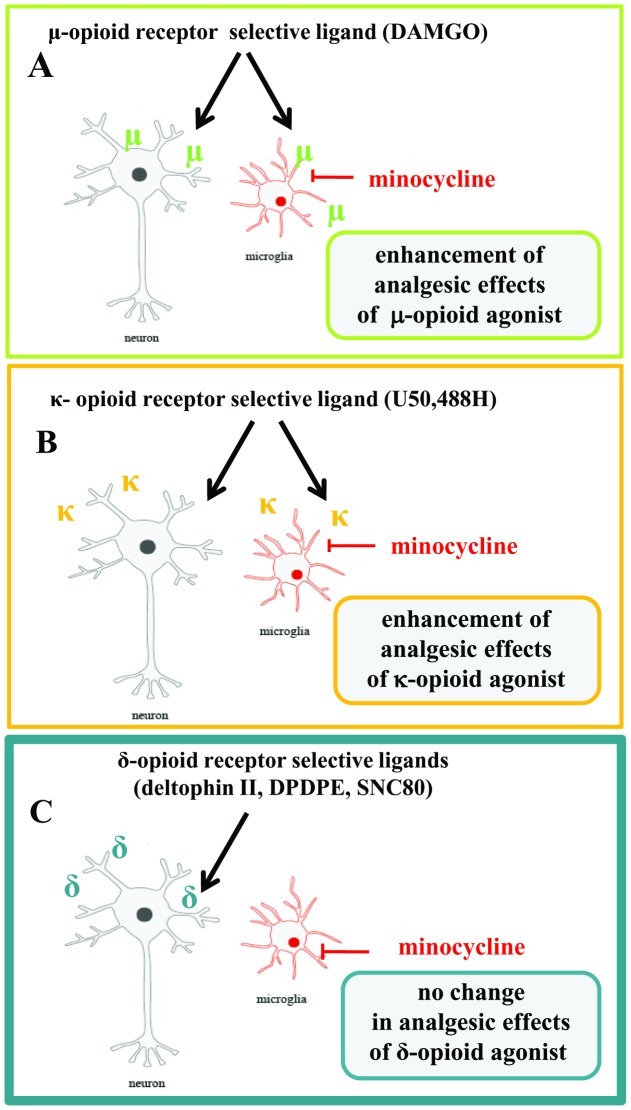
The possible influence of minocycline on analgesia after opioid receptors ligands. In our opinion, activated spinal microglia are key factors in the development of neuropathic pain and play a major role in the antagonizing of some opioids effectiveness. The results of our paper show for the first time that that DOR, in contrast to MOR and KOR, is not present in microglial cells. This phenomenon might be responsible for the different analgesic effects of MOR, KOR and DOR ligands (Fig. 2 and 3). We provide evidence that minocycline (a potent inhibitor of microglial activation and proliferation) enhances the effects of selective MOR (DAMGO; Fig. 2C,D) and selective KOR (U50,488H; Fig. 2E,F) agonists by inhibition of microglial cell activation. The effectiveness of DOR agonists (DPDPE, deltorphin II and SNC80) is not changed by minocycline (Fig. 3A–F). Our results indicate that an important element of the effectiveness of opioid drugs in neuropathic pain is the activation of microglia. The lack of DOR receptors in these cells causes that DOR receptor-mediated analgesia is not weaker under neuropathic pain, in which there is a strong activation of microglia. Earlier inhibition of microglial activation by minocycline administration therefore did not influence the effect of DOR selective agonists. The above results indicate not only that minocycline potentiates analgesia after MOR and KOR agonists but also that DOR is a potentially important target in the search for new drugs that would be effective against neuropathic pain.

The results of the present study document for the first time that DOR, in contrast to MOR and KOR, are not present in microglial cells (Figure 6). In conclusion, we provide evidence that minocycline not only diminishes neuropathic pain-related behaviour but also enhances the effectiveness of morphine and selective MOR and KOR opioid ligands under neuropathic pain conditions. Our findings lend support to the view that neuroimmunological changes in the spinal cord and DRG are important for opioid effectiveness in neuropathic pain. In our opinion, activated spinal microglia are key factors in not only the development of neuropathic pain but also in the different efficacies of opioid analgesics. Our results also suggest that DOR analgesia is not dependent on injury-induced microglial activation. We therefore suggest that DOR is an interesting target for the development of new drugs that would be effective against neuropathic pain.

## References

[1] Hirsch SJ, Dickenson AH (2014) Morphine sensitivity of spinal neurons in the chronic constriction injury neuropathic rat pain model. Neurosci Lett 562: 97–101. 24128881 10.1016/j.neulet.2013.10.007

[2] Przewlocki R, Przewlocka B (2001) Opioids in chronic pain. Eur J Pharmacol 429: 79–91. 11698029 10.1016/s0014-2999(01)01308-5

[3] Przewlocki R, Przewlocka B (2005) Opioids in neuropathic pain. Curr Pharm Des 11: 3013–3025. 16178760 10.2174/1381612054865055

[4] Eisenberg E, McNicol ED, Carr DB (2005) Efficacy and safety of opioid agonists in the treatment of neuropathic pain of non- malignant origin: Systematic review and meta-analysis of randomized controlled trials. JAMA 293: 3043–3052. 15972567 10.1001/jama.293.24.3043

[5] McQuay HJ (2002) Neuropathic pain: evidence matters. Eur J Pain 6: 11–8. 11888235 10.1053/eujp.2001.0316

[6] Evans CJ, Keith DE Jr, Morrison H, Magendzo K, Edwards RH (1992) Cloning of a delta opioid receptor by functional expression. Science 258: 1952–1955. 1335167 10.1126/science.1335167

[7] Kieffer BL, Befort K, Gavériaux-Ruff C, Hirth CG (1992) The delta-opioid receptor: isolation of a cDNA by expression cloning and pharmacological characterization. Proc Natl Acad Sci U S A 89: 12048–12052. 1334555 10.1073/pnas.89.24.12048 PMC50695

[8] Chen Y, Mestek A, Liu J, Yu L (1993) Molecular cloning of a rat kappa opioid receptor reveals sequence similarities to the mu and delta opioid receptors. Biochem J 295: 625–628. 8240267 10.1042/bj2950625 PMC1134603

[9] Minami M, Hosoi Y, Toya T, Katao Y, Maekawa K, et al (1993) In situ hybridization study of kappa-opioid receptor mRNA in the rat brain. Neurosci Lett 162: 161–164. 7907172 10.1016/0304-3940(93)90585-9

[10] George SR, Fan T, Xie Z, Tse R, Tam V, et al (2000) Oligomerization of mu- and delta-opioid receptors. Generation of novel functional properties. J Biol Chem 275: 26128–26135. 10842167 10.1074/jbc.M000345200

[11] Gomes I, Jordan BA, Gupta A, Trapaidze N, Nagy V, et al (2000) Heterodimerization of mu and delta opioid receptors: a role in opiate synergy. J Neurosci 20: RC110. 11069979 10.1523/JNEUROSCI.20-22-j0007.2000 PMC3125672

[12] Gomes I, Gupta A, Filipovska J, Szeto HH, Pintar JE, et al (2004) A role for heterodimerization of mu and delta opiate receptors in enhancing morphine analgesia. Proc Natl Acad Sci USA 101: 5135–5139. 15044695 10.1073/pnas.0307601101 PMC387386

[13] Mika J, Przewlocki R, Przewłocka B (2001) The role of delta-opioid receptor subtypes in neuropathic pain. Eur J Pharmacol 415: 31–37. 11245849 10.1016/s0014-2999(01)00814-7

[14] Nozaki C, Le Bourdonnec B, Reiss D, Windh RT, Little PJ, et al (2012) δ-Opioid mechanisms for ADL5747 and ADL5859 effects in mice: analgesia, locomotion, and receptor internalization. J Pharmacol Exp Ther 342: 799–807. 22700431 10.1124/jpet.111.188987 PMC3422521

[15] Obara I, Parkitna JR, Korostynski M, Makuch W, Kaminska D, et al (2009) Local peripheral opioid effects and expression of opioid genes in the spinal cord and dorsal root ganglia in neuropathic and inflammatory pain. Pain 141: 283–291. 19147290 10.1016/j.pain.2008.12.006

[16] Gavériaux-Ruff C, Kieffer BL (2011) Delta opioid receptor analgesia: recent contributions from pharmacology and molecular approaches. Behav Pharmacol 22: 405–414. 21836459 10.1097/FBP.0b013e32834a1f2c PMC3511815

[17] Fenalti G, Giguere PM, Katritch V, Huang XP, Thompson AA, et al (2014) Molecular control of δ-opioid receptor signalling. Nature 506: 191–196. 24413399 10.1038/nature12944 PMC3931418

[18] Somvanshi RK, Kumar U (2014) δ-Opioid Receptor and Somatostatin Receptor-4 Heterodimerization: Possible Implications in Modulation of Pain Associated Signaling. PLoS One 9: e85193. 24416361 10.1371/journal.pone.0085193 PMC3885706

[19] Lee YW, Chaplan SR, Yaksh TL (1995) Systemic and supraspinal, but not spinal, opiates suppress allodynia in a rat neuropathic pain model. Neurosci Lett 199: 111–114. 8584236 10.1016/0304-3940(95)12034-2

[20] Steele AD, Szabo I, Bednar F, Rogers TJ (2002) Interactions between opioid and chemokine receptors: heterologous desensitization. Cytokine Growth Factor Rev 13: 209–222. 12486875 10.1016/s1359-6101(02)00007-2

[21] Osikowicz M, Mika J, Przewlocka B (2013) The glutamatergic system as a target for neuropathic pain relief. Exp Physiol 98: 372–384. 23002244 10.1113/expphysiol.2012.069922

[22] Cui Y, Liao XX, Liu W (2008) A novel role of minocycline: attenuating morphine antinociceptive tolerance by inhibition of p38 MAPK in the activated spinal microglia. Brain Behav Immun 22: 114–123. 17919885 10.1016/j.bbi.2007.07.014

[23] Makuch W, Mika J, Rojewska E, Zychowska M, Przewlocka B (2013) Effects of selective and non-selective inhibitors of nitric oxide synthase on morphine- and endomorphin-1-induced analgesia in acute and neuropathic pain in rats. Neuropharmacology 75: 445–447. 24035921 10.1016/j.neuropharm.2013.08.031

[24] Mika J, Rojewska E, Makuch W, Przewlocka B (2010) Minocycline reduces the injury-induced expression of prodynorphin and pronociceptin in the dorsal root ganglion in a rat model of neuropathic pain. Neuroscience 165: 1420–1428. 19961904 10.1016/j.neuroscience.2009.11.064

[25] Mika J, Zychowska M, Popiolek-Barczyk K, Rojewska E, Przewlocka B (2013) Importance of glial activation in neuropathic pain. Eur J Pharmacol 716: 106–119. 23500198 10.1016/j.ejphar.2013.01.072

[26] Watkins LR, Maier SF (2003) Glia: a novel drug discovery target for clinical pain. Nat Rev Drug Discov 2: 973–985. 14654796 10.1038/nrd1251

[27] Wieseler-Frank J, Maier SF, Watkins LR (2005) Central proinflammatory cytokines and pain enhancement. Neurosignals 14: 166–174. 16215299 10.1159/000087655

[28] Amin AR, Attur MG, Thakker GD (1996) A novel mechanism of action of tetracyclines: effects on nitric oxide synthases. Proc Natl Acad Sci U S A 93: 14014–14019. 8943052 10.1073/pnas.93.24.14014 PMC19486

[29] Colovic M, Caccia S (2003) Liquid chromatographic determination of minocycline in brain-to-plasma distribution studies in the rat. Life Sci 791: 337–343. 10.1016/s1570-0232(03)00247-2 12798193

[30] Ledeboer A, Sloane EM, Milligan ED, Frank MG, Mahony JH, et al (2005) Minocycline attenuates mechanical allodynia and proinflammatory cytokine expression in rat models of pain facilitation. Pain 115: 71–83. 15836971 10.1016/j.pain.2005.02.009

[31] Pinkernelle J, Fansa H, Ebmeyer U, Keilhoff G (2013) Prolonged minocycline treatment impairs motor neuronal survival and glial function in organotypic rat spinal cord cultures. PLoS One 8: e73422. 23967343 10.1371/journal.pone.0073422 PMC3742532

[32] Tikka T, Fiebich BL, Goldsteins G, Keinanen R, Koistinaho J (2001) Minocycline, a tetracycline derivative, is neuroprotective against excitotoxicity by inhibiting activation and proliferation of microglia. J Neurosci 21: 2580–2508. 11306611 10.1523/JNEUROSCI.21-08-02580.2001 PMC6762519

[33] Niimi N, Kohyama K, Matsumoto Y (2013) Minocycline suppresses experimental autoimmune encephalomyelitis by increasing tissue inhibitors of metalloproteinases. Neuropathology 33: 612–616. 23581743 10.1111/neup.12039

[34] Machado LS, Kozak A, Ergul A, Hess DC, Borlongan CV, et al (2006) Delayed minocycline inhibits ischemia-activated matrix metalloproteinases 2 and 9 after experimental stroke. BMC Neurosci 7: 56. 16846501 10.1186/1471-2202-7-56 PMC1543649

[35] Liu X, Su H, Chu TH, Guo A, Wu W (2013) Minocycline inhibited the pro-apoptotic effect of microglia on neural progenitor cells and protected their neuronal differentiation in vitro. Neurosci Lett 542: 30–36. 23518153 10.1016/j.neulet.2013.03.011

[36] Garcia-Martinez EM, Sanz-Blasco S, Karachitos A, Bandez MJ, Fernandez-Gomez FJ, et al (2010) Mitochondria and calcium flux as targets of neuroprotection caused by minocycline in cerebellar granule cells. Biochem Pharmacol 79: 239–250. 19682437 10.1016/j.bcp.2009.07.028

[37] Kim TH, Kim HI, Kim J, Park M, Song JH (2011) Effects of minocycline on Na+ currents in rat dorsal root ganglion neurons. Brain Res 1370: 34–42. 21081117 10.1016/j.brainres.2010.11.038

[38] Jin SX, Zhuang ZY, Woolf CJ, Ji RR (2003) p38 mitogen-activated protein kinase is activated after a spinal nerve ligation in spinal cord microglia and dorsal root ganglion neurons and contributes to the generation of neuropathic pain. J Neurosci 23: 4017–4022. 12764087 10.1523/JNEUROSCI.23-10-04017.2003 PMC6741086

[39] Zhuang ZY, Wen YR, Zhang DR, Borsello T, Bonny C, et al (2006) A peptide c-Jun N-terminal kinase (JNK) inhibitor blocks mechanical allodynia after spinal nerve ligation: respective roles of JNK activation in primary sensory neurons and spinal astrocytes for neuropathic pain development and maintenance. J Neurosci 26: 3551–3560. 16571763 10.1523/JNEUROSCI.5290-05.2006 PMC6673862

[40] Chang YW, Waxman SG (2010) Minocycline attenuates mechanical allodynia and central sensitization following peripheral second-degree burn injury. J Pain 11: 1146–1154. 20418178 10.1016/j.jpain.2010.02.010

[41] Hains BC, Waxman SG (2006) Activated microglia contribute to the maintenance of chronic pain after spinal cord injury. J Neurosci 26: 4308–4317. 16624951 10.1523/JNEUROSCI.0003-06.2006 PMC6674010

[42] Hua XY, Svensson CI, Matsui T, Fitzsimmons B, Yaksh TL, et al (2005) Intrathecal minocycline attenuates peripheral inflammation-induced hyperalgesia by inhibiting p38 MAPK in spinal microglia. Eur J Neurosci 22: 2431–2440. 16307586 10.1111/j.1460-9568.2005.04451.x

[43] Radad K, Moldzio R, Rausch WD (2010) Minocycline protects dopaminergic neurons against long-term rotenone toxicity. Can J Neurol Sci 37: 81–85. 20169778 10.1017/s0317167100009690

[44] González JC, Egea J, Del Carmen Godino M, Fernandez-Gomez FJ, Sánchez-Prieto J, et al (2007) Neuroprotectant minocycline depresses glutamatergic neurotransmission and Ca(2+) signalling in hippocampal neurons. Eur J Neurosci 26: 2481–2495. 17986028 10.1111/j.1460-9568.2007.05873.x

[45] Mika J, Osikowicz M, Makuch W, Przewlocka B (2007) Minocycline and pentoxifylline attenuate allodynia and hyperalgesia and potentiate the effects of morphine in rat and mouse models of neuropathic pain. Eur J Pharmacol 560: 142–149. 17307159 10.1016/j.ejphar.2007.01.013

[46] Mika J, Wawrzczak-Bargiela A, Osikowicz M, Makuch W, Przewlocka B (2009) Attenuation of morphine tolerance by minocycline and pentoxifylline in naive and neuropathic mice. Brain Behav Immun 23: 75–84. 18684397 10.1016/j.bbi.2008.07.005

[47] Zychowska M, Rojewska E, Kreiner G, Nalepa I, Przewlocka B, et al (2013) Minocycline influences the anti-inflammatory interleukins and enhances the effectiveness of morphine under mice diabetic neuropathy. J Neuroimmunol 262: 35–45. 23870534 10.1016/j.jneuroim.2013.06.005

[48] Filipovic R, Zecevic N (2008) Neuroprotective role of minocycline in co-cultures of human fetal neurons and microglia. Exp Neurol 211: 41–51. 18359018 10.1016/j.expneurol.2007.12.024

[49] Kobayashi K, Imagama S, Ohgomori T, Hirano K, Uchimura K, et al (2013) Minocycline selectively inhibits M1 polarization of microglia. Cell Death Dis 4: e525. 23470532 10.1038/cddis.2013.54 PMC3613832

[50] Zimmermann M (1983) Ethical guidelines for investigations of experimental pain in conscious animals. Pain 16: 109–110. 6877845 10.1016/0304-3959(83)90201-4

[51] Bennett GJ, Xie Y (1988) A peripheral mononeuropathy in rat that produces disorders of pain sensation like those seen in man. Pain 33: 87–107. 2837713 10.1016/0304-3959(88)90209-6

[52] Osikowicz M, Mika J, Makuch W, Przewlocka B (2008) Glutamate receptor ligands attenuate allodynia and hyperalgesia and potentiate morphine effects in a mouse model of neuropathic pain. Pain 139: 117–126. 18442882 10.1016/j.pain.2008.03.017

[53] Yaksh TL, Rudy TA (1976) Chronic catheterization of the spinal subarachnoid space. Physiol. Behav 17: 1031–10. 14677603 10.1016/0031-9384(76)90029-9

[54] Zawadzka M, Kaminska B (2005) A novel mechanism of FK506-mediated neuroprotection: downregulation of cytokine expression in glial cells. Glia 49: 36–51. 15390105 10.1002/glia.20092

[55] Przanowski P, Dabrowski M, Ellert-Miklaszewska A, Kloss M, Mieczkowski J, et al (2014) The signal transducers Stat1 and Stat3 and their novel target Jmjd3 drive the expression of inflammatory genes in microglia. J Mol Med (Berl) 92: 239–254. 24097101 10.1007/s00109-013-1090-5 PMC3940857

[56] Chomczynski P, Sacchi N (1987) Single-step method of RNA isolation by acid guanidinium thiocyanate-phenol-chloroform extraction. Anal Biochem 162: 156–159. 2440339 10.1006/abio.1987.9999

[57] Mika J, Obara I, Przewlocka B (2011) The role of nociceptin and dynorphin in chronic pain: implications of neuro-glial interaction. Neuropeptides 45: 247–261. 21477860 10.1016/j.npep.2011.03.002

[58] Kerros C, Brood I, Sola B, Jauzac P, Allouche S (2010) Reduction of cell proliferation and potentiation of Fas-induced apoptosis by the selective kappa-opioid receptor agonist U50 488 in the multiple myeloma LP-1 cells. J Neuroimmunol 220: 69–78. 20163878 10.1016/j.jneuroim.2010.01.010

[59] Abcam website. Available: http://www.abcam.com/delta-opioid-receptor-antibody-ab66317.html. Accessed 2014 May 20.

[60] Neuromics website. Available: http://www.neuromics.com/ittrium/visit/A1x66x1y1x85b1x1x9cy1x6217x1x96y1x4fax1x82y1x510x1x7f. Accessed 2014 May 20.

[61] Mansson E, Bare L, Yang D (1994) Isolation of a human kappa opioid receptor cDNA from placenta. Biochem Biophys Res Commun 202: 1431–1437. 8060324 10.1006/bbrc.1994.2091

[62] Ko JL, Arvidsson U, Williams FG, Law PY, Elde R, et al (1999) Visualization of time-dependent redistribution of delta-opioid receptors in neuronal cells during prolonged agonist exposure. Brain Res Mol Brain Res 69: 171–185. 10366739 10.1016/s0169-328x(99)00094-7

[63] Mayer DJ, Mao J, Holt J, Price DD (1999) Cellular mechanisms of neuropathic pain, morphine tolerance, and their interactions. Proc Natl Acad Sci U S A 96: 7731–7736. 10393889 10.1073/pnas.96.14.7731 PMC33610

[64] Ossipov MH, Lopez Y, Nichols ML, Bian D, Porreca F (1995) The loss of antinociceptive efficacy of spinal morphine in rats with nerve ligation injury is prevented by reducing spinal afferent drive. Neurosci Lett 199: 87–90. 8584250 10.1016/0304-3940(95)12022-v

[65] Rowbotham MC, Twilling L, Davies PS, Reisner L, Taylor K, et al (2003) Oral opioid therapy for chronic peripheral and central neuropathic pain. N Engl J Med 13: 1223–1232. 10.1056/NEJMoa021420 12660386

[66] Gilron I, Bailey JM, Dongsheng Tu ME, Holden RR, et al (2005) Morphine, Gabapentin, or Their Combination for Neuropathic Pain. N Engl J Med 13: 1324–1334. 10.1056/NEJMoa042580 15800228

[67] Przewlocka B, Mika J, Labuz D, Toth G, Przewlocki R (1999) Spinal analgesic action of endomorphins in acute, inflammatory and neuropathic pain in rats. Eur J Pharmacol 367: 189–196. 10078992 10.1016/s0014-2999(98)00956-x

[68] Kabli N, Cahill CM (2007) Anti-allodynic effects of peripheral delta opioid receptors in neuropathic pain. Pain 127: 84–93. 16963185 10.1016/j.pain.2006.08.003

[69] Porreca F, Tang QB, Bian D, Riedl M, Elde R, et al (1998) Spinal opioid mu receptor expression in lumbar spinal cord of rats following nerve injury. Brain Res 795: 197–203. 9622629 10.1016/s0006-8993(98)00292-3

[70] Stone LS, Vulchanova L, Riedl MS, Williams FG, Wilcox GL, et al (2004) Effects of peripheral nerve injury on delta opioid receptor (DOR) immunoreactivity in the rat spinal cord. Neurosci Lett 361: 208–211. 15135930 10.1016/j.neulet.2003.12.067

[71] Herradon G, Ezquerra L, Nguyen T, Wang C, Siso A, et al (2008) Noradrenergic and opioidergic alterations in neuropathy in different rat strains. Neurosci Lett 438: 186–189. 18472331 10.1016/j.neulet.2008.03.095

[72] Pol O, Murtra P, Caracuel L, Valverde O, Puig MM, et al (2006) Expression of opioid receptors and c-fos in CB1 knockout mice exposed to neuropathic pain. Neuropharmacology 50: 123–132. 16360182 10.1016/j.neuropharm.2005.11.002

[73] Raghavendra V, Tanga F, Rutkowski MD, DeLeo JA (2003) Anti-hyperalgesic and morphine-sparing actions of propentofylline following peripheral nerve injury in rats: mechanistic implications of spinal glia and proinflammatory cytokines. Pain 104: 655–664. 12927638 10.1016/S0304-3959(03)00138-6

[74] Raghavendra V, Rutkowski MD, DeLeo JA (2002) The role of spinal neuroimmune activation in morphine tolerance/hyperalgesia in neuropathic and sham-operated rats. J Neurosci 22: 9980–9989. 12427855 10.1523/JNEUROSCI.22-22-09980.2002 PMC6757841

[75] Mika J, Korostynski M, Kaminska D, Wawrzczak-Bargiela A, Osikowicz M, et al (2008) Interleukin-1alpha has antiallodynic and antihyperalgesic activities in a rat neuropathic pain model. Pain 13: 587–597. 10.1016/j.pain.2008.02.015 18374486

[76] Cunningham CL, Martínez-Cerdeño V, Noctor SC (2013) Microglia regulate the number of neural precursor cells in the developing cerebral cortex. J Neurosci 33: 4216–4233. 23467340 10.1523/JNEUROSCI.3441-12.2013 PMC3711552

[77] Quock RM, Burkey TH, Varga E, Hosohata Y, Hosohata K, et al (1999) The delta-opioid receptor: molecular pharmacology, signal transduction, and the determination of drug efficacy. Pharmacol Rev 51: 503–532. 10471416

[78] Scherrer G, Imamachi N, Cao YQ, Contet C, Mennicken F, et al (2009) Dissociation of the opioid receptor mechanisms that control mechanical and heat pain. Cell 137: 1148–1159. 19524516 10.1016/j.cell.2009.04.019 PMC3683597

[79] Sluka KA, Rohlwing JJ, Bussey RA, Eikenberry SA, Wilken JM (2002) Chronic muscle pain induced by repeated acid Injection is reversed by spinally administered mu- and delta-, but not kappa-, opioid receptor agonists. J Pharmacol Exp Ther 302: 1146–1150. 12183674 10.1124/jpet.102.033167

[80] Holdridge SV, Cahill CM (2007a) Spinal administration of a delta opioid receptor agonist attenuates hyperalgesia and allodynia in a rat model of neuropathic pain. Eur J Pain 11: 685–693. 17175187 10.1016/j.ejpain.2006.10.008

[81] Holdridge SV, Armstrong SA, Taylor AM, Cahill CM (2007b) Behavioural and morphological evidence for the involvement of glial cell activation in delta opioid receptor function: implications for the development of opioid tolerance. Mol Pain 3: 7. 17352824 10.1186/1744-8069-3-7 PMC1828713

[82] Chang AC, Chao CC, Takemori AE, Gekker G, Hu S, et al (1996) Arylacetamide-derived fluorescent probes: synthesis, biological evaluation, and direct fluorescent labeling of kappa opioid receptors in mouse microglial cells. J Med Chem 39: 1729–1735. 8648612 10.1021/jm950813b

[83] El-Hage N, Dever SM, Podhaizer EM, Arnatt CK, Zhang Y, et al (2013) A novel bivalent HIV-1 entry inhibitor reveals fundamental differences in CCR5-μ-opioid receptor interactions between human astroglia and microglia. AIDS 27: 2181–2190. 23751259 10.1097/QAD.0b013e3283639804 PMC3918492

[84] Merighi S, Gessi S, Varani K, Fazzi D, Stefanelli A, et al (2013) Morphine mediates a proinflammatory phenotype via μ-opioid receptor-PKCε-Akt-ERK1/2 signaling pathway in activated microglial cells. Biochem Pharmacol 86: 487–496. 23796752 10.1016/j.bcp.2013.05.027

[85] Chao CC, Gekker G, Hu S, Sheng WS, Shark KB, et al (1996) Kappa opioid receptors in human microglia downregulate human immunodeficiency virus 1 expression. Proc Natl Acad Sci U S A 93: 8051–8056. 8755601 10.1073/pnas.93.15.8051 PMC38873

[86] Chao CC, Hu S, Shark KB, Sheng WS, Gekker G, et al (1997) Activation of mu opioid receptors inhibits microglial cell chemotaxis. J Pharmacol Exp Ther 281: 998–1004. 9152411

[87] Turchan-Cholewo J, Dimayuga FO, Ding Q, Keller JN, Hauser KF, et al (2008) Cell-specific actions of HIV-Tat and morphine on opioid receptor expression in glia. J Neurosci Res 86: 2100–2110. 18338799 10.1002/jnr.21653 PMC2760290

[88] Thorlin T, Persson PA, Eriksson PS, Hansson E, Rönnbäck L (1999) Delta-opioid receptor immunoreactivity on astrocytes is upregulated during mitosis. Glia 25: 370–378. 10028919 10.1002/(sici)1098-1136(19990215)25:4<370::aid-glia6>3.0.co;2-j

[89] Scherrer G, Tryoen-Tóth P, Filliol D, Matifas A, Laustriat D, et al (2006) Knockin mice expressing fluorescent delta-opioid receptors uncover G protein-coupled receptor dynamics in vivo. Proc Natl Acad Sci U S A 103: 9691–9696. 16766653 10.1073/pnas.0603359103 PMC1480468

[90] Commons KG, Milner TA (1996) Cellular and subcellular localization of delta opioid receptor immunoreactivity in the rat dentate gyrus. Brain Res 738: 181–195. 8955512 10.1016/s0006-8993(96)00774-3

[91] Cheng PY, Liu-Chen LY, Pickel VM (1997) Dual ultrastructural immunocytochemical labeling of mu and delta opioid receptors in the superficial layers of the rat cervical spinal cord. Brain Res 778: 367–380. 9459554 10.1016/s0006-8993(97)00891-3

[92] Horvath RJ, DeLeo JA (2009) Morphine enhances microglial migration through modulation of P2X4 receptor signaling. J Neurosci 29: 998–1005. 19176808 10.1523/JNEUROSCI.4595-08.2009 PMC2727471

[93] Kieffer BL, Gavériaux-Ruff C (2002) Exploring the opioid system by gene knockout. Prog Neurobiol 66: 285–306. 12015197 10.1016/s0301-0082(02)00008-4

[94] Gavériaux-Ruff C, Karchewski LA, Hever X, Matifas A, Kieffer BL (2008) Inflammatory pain is enhanced in delta opioid receptor-knockout mice. Eur J Neurosci 27: 2558–2567. 18513322 10.1111/j.1460-9568.2008.06223.x PMC4445739

[95] Nadal X, Baños JE, Kieffer BL, Maldonado R (2006) Neuropathic pain is enhanced in delta-opioid receptor knockout mice. Eur J Neurosci 23: 830–834. 16487163 10.1111/j.1460-9568.2006.04569.x

[96] Gallantine EL, Meert TF (2005) A comparison of the antinociceptive and adverse effects of the mu-opioid agonist morphine and the delta-opioid agonist SNC80. Basic Clin Pharmacol Toxicol 97: 39–51. 15943758 10.1111/j.1742-7843.2005.pto_97107.x

[97] Cahill CM, Morinville A, Hoffert C, O'Donnell D, Beaudet A (2003) Up-regulation and trafficking of delta opioid receptor in a model of chronic inflammation: implications for pain control. Pain 101: 199–208. 12507715 10.1016/s0304-3959(02)00333-0

[98] Fraser GL, Gaudreau GA, Clarke PB, Ménard DP, Perkins MN (2000) Antihyperalgesic effects of delta opioid agonists in a rat model of chronic inflammation. Br J Pharmacol 129: 1668–1672. 10780972 10.1038/sj.bjp.0703248 PMC1572005

[99] Hurley RW, Hammond DL (2000) The analgesic effects of supraspinal mu and delta opioid receptor agonists are potentiated during persistent inflammation. J Neurosci 20: 1249–1259. 10648729 10.1523/JNEUROSCI.20-03-01249.2000 PMC6774182

[100] Happel C, Steele AD, Finley MJ, Kutzler MA, Rogers TJ (2008) DAMGO-induced expression of chemokines and chemokine receptors: the role of TGF-beta1. J Leukoc Biol 83: 956–963. 18252865 10.1189/jlb.1007685

[101] Szabo I, Chen XH, Xin L, Adler MW, Howard OM, et al (2002) Heterologous desensitization of opioid receptors by chemokines inhibits chemotaxis and enhances the perception of pain. Proc Natl Acad Sci U S A 99: 10276–10281. 12130663 10.1073/pnas.102327699 PMC124904

[102] Horvath RJ, Romero-Sandoval EA, De Leo JA (2010b) Inhibition of microglial P2X4 receptors attenuates morphine tolerance, Iba1, GFAP and μ opioid receptor protein expression while enhancing perivascular microglial ED2. Pain 150: 401–413. 20573450 10.1016/j.pain.2010.02.042 PMC2921455

[103] Hutchinson MR, Coats BD, Lewis SS, Zhang Y, Sprunger DB, et al (2008) Proinflammatory cytokines oppose opioid-induced acute and chronic analgesia. Brain Behav Immun 22: 1178–1189. 18599265 10.1016/j.bbi.2008.05.004 PMC2783238

[104] Cahill CM, Holdridge SV, Morinville A (2007) Trafficking of delta-opioid receptors and other G-protein-coupled receptors: implications for pain and analgesia. Trends Pharmacol Sci 28: 23–31. 17150262 10.1016/j.tips.2006.11.003

[105] Bie B, Pan ZZ (2007) Trafficking of central opioid receptors and descending pain inhibition. Mol Pain 3: 37. 18053223 10.1186/1744-8069-3-37 PMC2219988

[106] Zhang X, Bao L, Guan JS (2006) Role of delivery and trafficking of delta-opioid peptide receptors in opioid analgesia and tolerance. Trends Pharmacol Sci 27: 324–329. 16678916 10.1016/j.tips.2006.04.005

[107] Pradhan AA, Befort K, Nozaki C, Gavériaux-Ruff C, Kieffer BL (2011) The delta opioid receptor: anevolving target for the treatment of braindisorders. Trends Pharmacol Sci 32: 581–590. 21925742 10.1016/j.tips.2011.06.008 PMC3197801

[108] Clinicaltrials website. Available: http://clinicaltrials.gov/ct2/results?term=delta+opioid&Search=Search. Accessed 2014 May 20.

